# Single-nucleus profiling of adult mice sub-ventricular zone after blast-related traumatic brain injury

**DOI:** 10.1038/s41597-022-01925-y

**Published:** 2023-01-05

**Authors:** Manrui Li, Xiameng Chen, Qiuyun Yang, Shuqiang Cao, Steven Wyler, Ruixuan Yuan, Lingxuan Zhang, Miao Liao, Meili Lv, Feng Wang, Yadong Guo, Jihong Zhou, Lin Zhang, Xiaoqi Xie, Weibo Liang

**Affiliations:** 1 Shanghai Key Lab of Forensic Medicine, Key Lab of Forensic Science, Ministry of Justice, Shanghai, 200000 China; 2 Department of Forensic Genetics, West China School of Basic Medical Sciences and Forensic Medicine, Sichuan University, Chengdu, 610041 China; 3 Department of Forensic Pathology and Forensic Clinical Medicine, West China School of Basic Medical Sciences and Forensic Medicine, Sichuan University, Chengdu, 610041 China; 4 Department of Internal Medicine, UT Southwestern Medical Center, Dallas, TX 75390 USA; 5 Sichuan University, Chengdu, 610041 China; 6 Department of Immunology, West China School of Basic Medical Sciences and Forensic Medicine, Sichuan University, Chengdu, 610041 China; 7 Department of Medical Oncology, Cancer Center, Sichuan University, Chengdu, 610041 China; 8 Department of Forensic Science, School of Basic Medical Sciences, Central South University, Changsha, Hunan 410013 China; 9 Army Medical University, Chongqing, 404000 China; 10 Department of Critical Care Medicine, Sichuan University, Chengdu, 610041 China

**Keywords:** White matter injury, Pathogenesis

## Abstract

Explosive blast-related traumatic brain injuries (bTBI) are common in war zones and urban terrorist attacks. These bTBIs often result in complex neuropathologic damage and neurologic complications. However, there is still a lack of specific strategies for diagnosing and/or treating bTBIs. The sub-ventricular zone (SVZ), which undergoes adult neurogenesis, is critical for the neurological maintenance and repair after brain injury. However, the cellular responses and mechanisms that trigger and modulate these activities in the pathophysiological processes following bTBI remain poorly understood. Here we employ single-nucleus RNA-sequencing (snRNA-seq) of the SVZ from mice subjected to a bTBI. This data-set, including 15272 cells (7778 bTBI and 7494 control) representing all SVZ cell types and is ideally suited for exploring the mechanisms underlying the pathogenesis of bTBIs. Additionally, it can serve as a reference for future studies regarding the diagnosis and treatment of bTBIs.

## Background & Summary

Explosive blasts often cause blast-related traumatic brain injury (bTBI), which is common among military service members and veterans during combat and training exercises^1^. Although the burden of global armed conflict has decreased in the past few decades, terrorist attacks and regional conflicts still occur frequently. Currently, the Military Acute Concussion Evaluation (MACE) and Automated Neuropsychological Assessment Metrics (ANAM) tests are used to assess possible bTBI and its associated neurocognitive deficits^2^. However, these subjective ratings may not accurately reflect the degree of brain injury. Also, routine imagological examination such as CT and MRI may not be able to reveal lesions following a bTBI, thus leaving the diagnosis of bTBI an issue to be addressed as there are affected areas which are not observed in the imagological examination. Additionally, the pathogenesis post bTBI has not been fully elucidated, and there is no specific and evidence-based treatment for bTBI patients^3^.

Studies have shown that sub-ventricular zone (SVZ), an important neurogenic niche in the adult rodent brain^4^, participates in the pathophysiological process after traumatic brain injury (TBI) at different stages^5^. The SVZ is located at the border of lateral cerebral ventricles, it harbors neural stem cells (NSCs) and gives rise to new neural cells^6^. In physiological status, the SVZ-generated neurons migrate along the rostral migratory stream to supply the newborn neurons in the olfactory bulb^7^. Upon brain injury, NSCs in the SVZ respond by increasing cell proliferation, differentiation and migration to the injured areas^8^. The bTBI induced pathogenesis differs from that of other types of TBIs^9^. However it remains unclear how various SVZ cell populations respond to the bTBI induced cell degeneration and cell death, as well as the underlying mechanisms. Identifying the transcriptomic changes at a single cell resolution will provide an extensive resources for the study of mechanisms underlying bTBI induced pathogenesis, and should aid in the development of specific diagnostic methods and/or treatments.

In recent years, the development of single-cell RNA-sequencing (scRNA-seq) and single-nucleus RNA-sequencing (snRNA-seq) has facilitated the discovery of cell-type specific markers, the understanding of cell sub-populations and heterogeneities, and the profiling of cell type specific gene expression, in a high throughput manner. It has the advantage over traditional bulk RNA-seq which represents the average signal of gene expression across the cell populations whereas, scRNA-seq can capture cellular details which could not otherwise be resolved using bulk RNA-seq. Therefore, we employed this powerful tool to generate a snRNA-seq data-set in this study, to achieve a high resolution of bTBI induced transcriptome profiling of the SVZ neurogenesis niche.

This data-set provides an unbiased transcriptional atlas of cell populations from control and bTBI mice SVZ representing all known SVZ cell types at sufficient levels needed for a deep analysis of the SVZ populations. These data can be used to cluster SVZ cells, explore novel cell markers, analyze transcriptome characteristics and predict the lineage trajectory of NSCs in response to bTBI. This data-set is thus suited for use by those interested in exploring the molecular mechanisms of bTBI induced pathogenesis, as well as those wishing to use it as a resource for discovering specific targets for the diagnosis or treatment of bTBIs.

## Methods

### Animals and treatments

7-week-old C57BL/6 J male mice (weight, 20–22 g) were obtained from ENSIWEIER Bio-Technology Co., Ltd. (Chengdu, China) and were maintained in an animal facility in the Experimental Center of Medical Animal of the Daping Hospital, the Third Military Medical University at 12 h/12 h dark/light cycle, temperature 22 °C, humidity 50%. Animals have free access to water and standard rodent chow. All procedures on animals meet the local laws and institutional guidelines (APPROVAL NUMBER: 2020386 A), and were performed under the guide for the Care and Use of Laboratory Animals of NIH (NIH publication #85-23, revised in 1985).

Animals were randomly divided into 2 groups- a bTBI group and Sham group, with 3 mice included in each group. The number of animals used in this experiment was determined according to a previous study performed by Arneson, D *et al*.^10^. Anesthesia was induced in mice with intraperitoneal injection of pentobarbital (5 mg/100 g ip). For bTBI mice, a compressed gas-driven BST-I bio-shock tube apparatus was used to generate blast wave, thereby inducing a bTBI^11^. The apparatus is driven by high-pressure compressed gas which breaks an aluminum sheet and generate blast waves similar to those found in open-field conditions. The intensity and duration of the blast wave can be adjusted by the position of the aluminum sheet, which guarantees the stability and controllability of blast wave. Mice are confined in small individual compartments that restrict their movement, with their head in the direction of the blast wave. 5.0 MPa explosion generated blast waves which can be directly measured by a pressure transducer set beside the mice. 48 h after injury, the mice were anesthetized and perfused with PBS. Then the SVZ were dissected and retrieved using the protocol described by Walker T L *et al*.^12^. The tissue samples were kept in MACS Tissue Storage Solution (Miltenyi Biotec) until processing. The Sham mice received anesthesia only.

### Validation of brain injury

To evaluate the degree of nerve injury and behavior functional deficits, we performed behavior experiments on mice 6 h after BE based on the criteria of modified neurological severity score (mNSS). Ten behavioral tasks were administrated on mice to observe the motor function, alertness, balancing, and general behavior of mice after blast exposure. Behavioral tasks and corresponding ratings are given in the previous study^13^. mNSS provides a simple method to detect multiple deficits after brain injury, and the mNSS scale is a commonly used approach for assessing the degree of neural impairments in mice and deficits could be marked by the composite score. The extent of injury is described as follows: the composite mNSS score of 3–4, 5–6 and 7–8 are for mild, moderate and severe TBI respectively. while 9–10 is regarded as lethal injury^14^.

### Nucleus isolation

Free RNAs released from dead cells in the single cell suspension would lead to noise in the data, and this background effect may affect the data quality and sequencing accuracy. Thus, we introduced a tissue- specific enzymatic digestion to prepare viable single-cell suspension to minimize the number of dead cells. Nuclei were isolated and purified from fresh tissue, as described previously^15^. Briefly, the frozen tissue was homogenized in NLB buffer which contain 250 mM Sucrose, 10 mM Tris-HCl, 3 mM MgAc2, 0.1% Triton X-100 (SigmaAldrich, USA), 0.1 mM EDTA, 0.2U/μL RNase Inhibitor (Takara, Japan). As a widely used method to purify nuclei from brain cells, ultracentrifugation through discontinuous sucrose gradients is then used to further purify the nuclei. The concentration of nucleus was adjusted to about 1000 nuclei/μL for snRNA-seq.

### 10×Genomics snRNA-seq experiment

10 × Genomics library preparation and single-nucleus RNA-Seq were performed by NovelBio Co.,Ltd (Shanghai, China) using the 10 × Genomics Chromium Controller Instrument and Chromium Single Cell 3′ V3 Reagent Kits (10 × Genomics, Pleasanton, USA). After washing twice in PBS, nuclei were incubated for 30 min at room temperature and then approximately 1000 nuclei/μL were loaded into 10 × Chromium chips, which has been already loaded with 10 × single cell 3 ‘V3 chemistry and barcode, to generate single-cell Gel Bead-In-Emulsions (GEMs). After the synthesis of cDNA, GEMs were broken and barcoded-cDNA was amplified for 14 cycles after library construction. Following fragmentation and index PCR amplification, the final libraries were quantified using the Qubit High Sensitivity DNA assay (Thermo Fisher Scientific, USA) and the size distribution of the libraries was determined using a High Sensitivity DNA chip on a Bioanalyzer 2200 (Agilent, USA). All libraries were sequenced by Novaseq. 6000 platform (Illumina, USA) with a 2 × 150 bp paired- end sequencing protocol. We detected over 726 M reads in total, of which 47589 reads were detected per cell.

### Alignment of reads to transcripts and cells

We applied fastp with default parameter to filter the adaptor sequence and removed the low-quality reads to achieve the clean data. CellRanger v3.1.0 was used to align the short reads to the mouse reference genome (GRCm38 Ensembl: version 92), to obtain feature-barcode matrices, including valid cell barcode and UMI count of transcripts. RNA-seq analysis was performed based on the confidently mapped reads with cell-associated barcodes, and an aggregated matrix was obtained by counting UMIs for each gene. To better guarantee the data quality, we excluded cells with less than 200 detected genes or more than 10% mitochondrial UMIs rate. Notably, mitochondrial genes were removed in the analysis.

### PCA, t-SNE and UMAP analysis

The Cell Ranger Software and Seurat packages were used to perform data analysis. The UMI-based clean data (scaled data) were obtained by the Seurat package based on the UMI counts of each sample and the percentage of mitochondrial rate for cell normalization and regression. t-SNE and UMAP were constructed by performing PCA based on top 2000 highly variable genes and top 10 principals from the scaled data. A graph-based, unsupervised clustering approach is introduced to cluster cells according to the top 10 principal components. Marker genes are automatedly calculated by Wilcoxon rank-sum test using Seurat’s FindAllMarkers function, and filtered under following criteria: 1. lnFC > 0.25; 2. p < 0.05; 3. min.pct >0.1. To further characterize sub-types of cells, we re-analyzed the gene data within the same cell type for graph-based clustering and marker analysis.

### Pseudo-time analysis

Neural stem cell (NSC) trajectories analysis was applied utilizing Monocle2 (http://cole-trapnell-lab.github.io/monocle-release). Before Monocle analysis, all detected NSCs data including markers genes in each cluster and raw expression counts were selected by Seurat. Based on the pseudo-time analysis, branched expression analysis modeling (BEAM) analysis was applied to analyze branch fate determined genes over a single-cell trajectory. Top 50 most significantly differentially expressed genes were used to generate fate-determined gene heatmap within different cell branches.

## Data Records

Raw data are accessible in the Gene Expression Omnibus (project number: GSE207078 for bTBI (GSM6276819)^16^; project number: GSE198074 for Sham (GSM5938054)^17^. Expression matrices, UMI counts for each gene and in each cell have been deposited in Figshare as matrix.mtx.gz, features.tsv.gz and barcodes.tsv.gz in a matrix format, respectively (10.6084/m9.figshare.20174240.v2 for bTBI^18^; 10.6084/m9.figshare.20174261.v1 for Sham)^19^. The related cell and gene information are contained in these matrices files, with the identifiers of columns and rows contained included in TSV files. These files correspond to the FASTQ data produced by CellRanger pipeline.

## Technical Validation

**Fig. 1:**
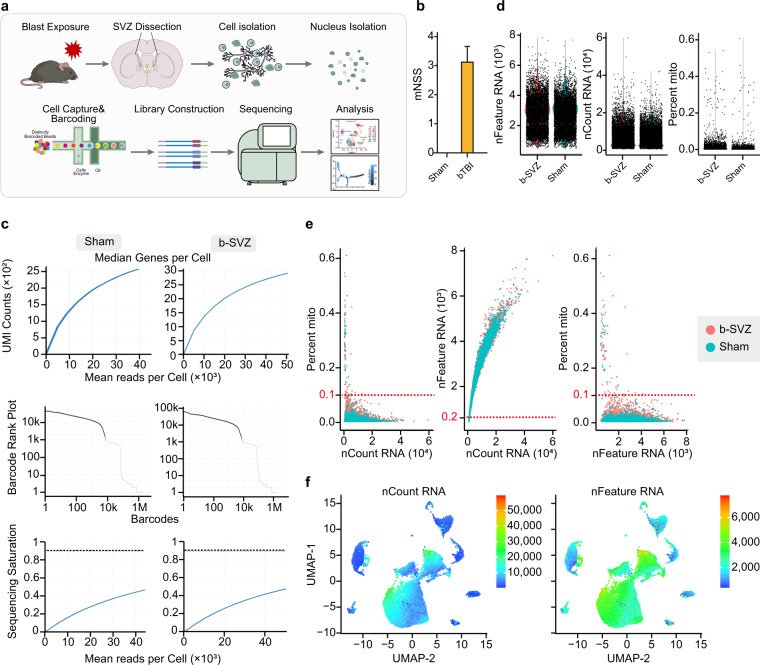
Quality control (QC) of mice SVZ snRNA-seq data. (**a**) Schematic representation of the experimental workflow. (**b**) mNSS score of bTBI and Sham mice. (**c**) Curves showing sequencing depth from bTBI and Sham SVZ, upper: relationship between mean reads per cell and median genes per cell, middle: relationship between barcodes and unique molecular identifier (UMI) counts, lower: relationship between mean reads per cell and sequencing saturation. (**d**) Scatterplot illustrating the number of detected genes (left), UMIs (middle), and the percentage of mitochondrial gene (right) in each cell of the two samples. (**e**) Scatterplot showing the correlation of the percentage of mitochondrial genes and the detected mRNA counts (UMIs) (left), the gene counts and the detected mRNA counts (UMIs) (middle), together with the percentage of mitochondrial genes and the gene counts (right). The lines in the plot representing the thresholds used at the cell filtration step. (**f**) The mRNA count (left) and the gene count (right) mapped on to the UMAP projection.

We constructed the snRNA-seq library using the 10 × Genomics method, which was sequenced on an Illumina NovelSeq. 6000 platform (Fig. 1a). The SVZ derived single-cell suspension was obtained from bTBI mice and sham mice (Fig. 1b). The saturation curve analysis showed that the data quality metrics of bTBI group and sham group (Fig. 1c, Table 1) were comparable and were sufficient to detect the highest number of expressed genes in each cell. The detailed sequencing depth data were listed in Table 1. The total reads in both groups were more than 332 M. The number of valid barcodes detected was 96.5%. (Table 2). Median genes and Mean reads measured in each cell were 2703 and 47589, respectively, and the batch effect of samples was minimized by Seurat algorithm. The sequencing quality of the two groups was high and comparable, indicating little technical bias introduced in the study. Collectively, we generated a high-quality single-nucleus transcriptome data-set of adult mice SVZ from a bTBI group and sham group.

**Table 1:** Sequencing and Cell Ranger statistics.

Sample	Estimated Number of Cells	Fraction Reads in Cells(%)	Mean Reads per Cell	Median Genes per Cell	Total Genes Detected	Median UMI Counts per Cell	Sequencing Saturation (%)
bTBI	7778	57	50690	2912	23899	7844	47.9
Sham	7494	59.6	44371	2663	23504	6756	46.6

**Table 2:** Detailed QC of FASTQ files.

Sample	bTBI	Sham
Number of Reads(K)	394268	332514
Valid Barcodes(%)	96.9	96.5
Valid UMI(%)	100	99.9
Q30 Bases in RNA Reads(%)	92.4	91.3
GC Content(%)	43.137	42.442
Reads Mapped Confidently to Genome(%)	92.9	91.9
Reads Mapped Confidently to Exonic Region(%)	85.8	84.9
Reads Mapped Confidently to Transcriptome(%)	59.3	54.2

The statistical metrics of sequencing data at single cell level were displayed in Table 2 and Fig. 1c. The estimated number of cells detected in bTBI group and sham group were 7778 and 7494, the median UMI detected in each cell were 7844 and 6756, the mean reads per cell were 50690 and 44371, and the average gene counts was 2912 and 2663, respectively. These metrics were equal between the two groups, indicating a fair proportionality of our sequencing data. Therefore, it is suited to perform the transcriptomic analysis of both groups using these sequencing data. In addition, according to the official guidelines of the 10 × Genomics Single Cell 3′ V3, gene expression libraries require a sequencing depth of more than 20 K reads per Cell. To acquire more information of gene expression, we selected a sequencing depth of 50 K reads per cell. Therefore, our sequencing depth was sufficient for subsequent analysis.

In order to ensure that all the barcodes correspond to viable cells, we screened cells using the percentage of mitochondrial transcription products (percent.mito) and the counts of genes detected. For necrotic and apoptotic cells, cell membrane ruptures and its permeability increases, leading to extravasation of cytoplasmic RNA, while mitochondrial transcripts are retained, hence we interpreted high percent.mito as necrotic and apoptotic cells (Fig. 1d). We set the threshold- percent.mito <0.1 to roughly remove necrotic and apoptotic cells in the data (Fig. 1e). In addition, cells with less than 200 detected genes are regarded as necrotic or apoptotic cells, and gene counts >10000 per cell was interpreted as doublet, both of which has been removed from our data (Fig. 1e). Through the above screening, most of the cells were retained, indicating that our sample preparation and sequencing process were of high quality and the data were reliable.

**Fig. 2:**
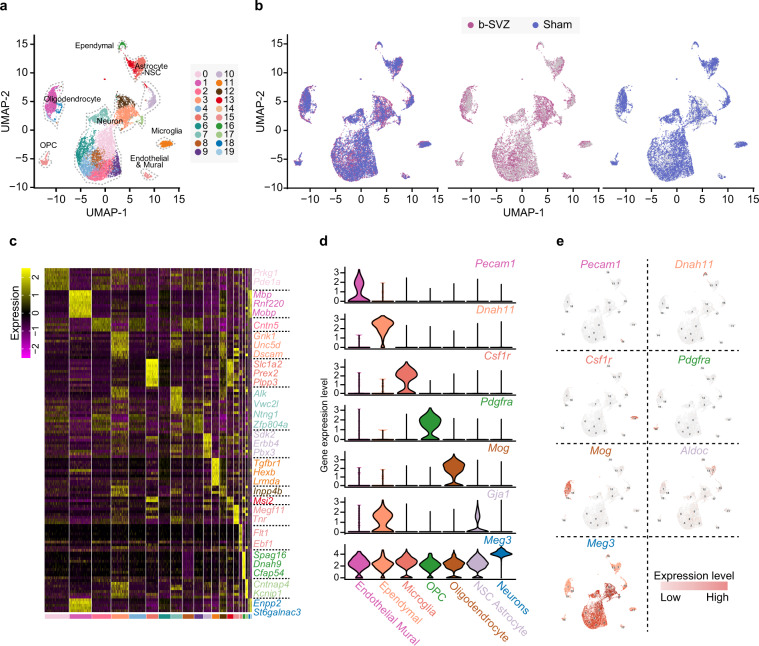
snRNA-seq reveals the cell heterogeneity within the SVZ of mice. (**a**) UMAP overview of automatic unsupervised clustering, 19 clusters in total. Clusters were defined into cell type by cell specific markers (Table 3). (**b**) UMAP projections of SVZ cells from bTBI and Sham mice. (**c**) Heatmap showing the top detected markers associated with the 7 major cell types identified. With selected neuronal cell function associated maker genes annotated on the right. (**d**) Violin-distributions of known maker genes expressed by each cell type. (**e**) Relative expression of gene characteristic for each cell type.

To dissect the cell populations and further validate the application value of these data, we used principal component analysis (PCA) and UMAP (Fig. 2a) projection to reduce and present the feature dimensions of the two groups. Under unsupervised calculation, 19 clusters were resolved. Of note, there was little divergence in the cell distribution of the two groups (Fig. 2b), which indicated the consistency of the sequencing data from the two groups.

To dissect the major cell types in SVZ, we conducted unsupervised clustering based on featured gene expression. We obtained the single-cell marker gene population through novel differential expression analysis algorithm^20^. Based on these marker genes, we identified the cell type of each population, which further supported the application value of these data. Based on known cell type markers (Table 3), SVZ cells were divided into 7 major populations: neurons, neural stem cell (NSC)- astrocytes, oligodendrocytes, OPC, microglias, ependymal cells and endothelium- mural cells (Fig. 2b). The neuron specific marker- *Meg3* was used to define 11 clusters as neurons^21^. In addition, genes highly expressed in neurons such as *Syt1* and *Snap25* are highly enriched in those clusters, which further supported our identification^22^. Cluster11 specifically expresses *Csf1r*, therefore defined as microglia^23^. Additionally, Cluster 11, had high enrichment of *Tmem119* and *P2ry12* common microglial markers. The SVZ niche astrocytes have long been believed to function as NSCs, and share many hallmarks with the NSCs^8,24^. Hence, cluster 5 and cluster 13, which highly expressed astrocyte&NSC marker- *Nr2e1*,*Hes5* and *Slc1a2*, were grouped together for analysis^8^. Myelin oligodendrocyte glycoprotein (*Mog*) and *Aspa* are specific marker of oligodendrocyte lineage cells and mature oligodendrocytes respectively^25,26^. Cluster1, 18, 19 were defined as oligodendrocytes as they highly expressed these two markers. Genes highly expressed in oligodendrocytes in previous publications (such as *Tubb4a* and *Apod*)^22^ were also found to be enriched in Cluster1, 18, 19. Cluster14 was defined as oligodendrocyte precursor cells (OPCs) by the specific marker *Pdgfra*^22^. Cluster16 was identified to be ependymal cells according to its exclusive expression of *Dnah11*^21^. Other enriched genes of ependymal cells- *Spag16*,*Adamts20*, *Ak7*,*Ak9*, *Armc3*, are highly expressed in cluster16^21^. As both endothelial markers- Platelet endothelial cell adhesion molecule 1 (*Pecam1*), Von Willebrand factor (*Vwf*) and mural cell marker- vitriendin (*Vtn*) were found to be specifically enriched in cluster15, we defined cluster15 as endothelial- mural cell^21,27^ (Fig. 2c–e).

**Table 3:** Known cell type marker genes of major cell clusters.

Cell type	Markers
Neuron	Meg3, Syt1, Snap25
Microglia	Csf1r, Tmem119, P2ry12
NSC-Astrocyte	Slc1a3, Apoe, Aldoc, Aqp4
Oligodendrocyte	Mog, Aspa, Tubb4a, Apod
OPC	Pdgfra
Ependymal	Dnah11, Spag16,Adamts20, Ak7,Ak9, Armc3
Endothelial-Mural	Pecam1(CD31), Vwf, Vtn

**Fig. 3:**
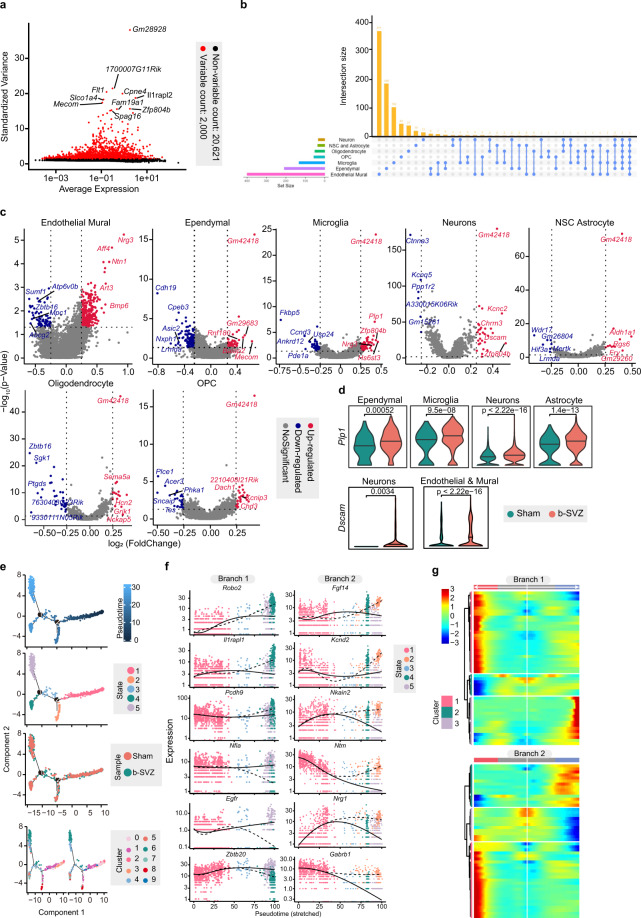
Differential expression gene (DEGs) analysis and single cell trajectory analysis. (**a**) Scatterplot revealing the relationship between gene dispersion and average expression of each gene, illustrating the difference between each cell in snRNA seq. (**b**) DEGs UpSet Plot. The bar on the left showing the number of DEGs for each cell type. The internal bar showing the number of DEGs unique and shared by each cell type and the related cell types are indicated by the bubble plot below. (**c**) Volcano plot showing DEGs in different cell types. Red dots indicate upregulated genes, blue dots indicate down-regulated genes, with gene names annotated next to the corresponding dots. (**d**) Violin plots representing the expression of Plp1 and Dscam in correlating cell types of bTBI and Sham mice. (**e**) Monocle2-generated pseudo-temporal trajectory of NSC-Astrocyte. Two distinct branch points were identified by Monocle2. At branch point, cells must choose a certain gene expression program. Cells on the tree are coloured based on pseudo-time in a gradient from dark to light blue, and the start of pseudo-time is indicated by dark blue, the end of pseudo-time is indicated by light blue (upper). The pseudo-time trajectory was divided into 5 different states by Monocle2 and are coloured respectively (upper middle). The trajectory showing the distribution of cells (lower middle) and the distribution of the 9 sub-classes of NSC-Astrocyte (lower) from two samples. (**f**) Line plots showing the top six cell fate related genes as the expression level over pseudo-time by Monocle2. (**g**) Heatmap for clustering the top 50 cell fate related genes at each branch point. These genes were divided into three clusters, showing genes at the beginning stage, the transitory stage and the end stage of the differentiation trajectory, respectively.

To further validate the value of our sequencing data in uncovering transcriptomic alterations following bTBI, we performed a differential expression gene (DEGs) analysis. Combining FindMarkers and the Wilcox rank-sum test, 910 significant DEGs were screened out under a predefined standard (1. lnFC > 0.25; 2. P < 0.05; 3. Min. PCT > 0.1) (Fig. 3a). By processing the data using R Package UpSetR, the Regression Interpreting Residual Plots of DEGs were mapped (Fig. 3b). Among the various types of cells, most DEGs (≥80%) of neurons, NSC-astrocytes, and endothelium-mural cells were up-regulated, suggesting that bTBI may promote the activation of these cell functions. In oligodendrocytes and ependymal cells, a large number of DEGs (≥70%) were down-regulated, indicating a relatively depressed state. Interestingly, we found that a large part of DEGs were exclusively changed only in endothelium-mural cells (n = 371), ependymal cells (n = 185), or microglia (n = 102), suggesting that these DEGs have a strong cell-type specificity (Fig. 3b). Functional analysis of the DEGs was then performed to act as a biological quality control of our data. For instance, studies have found that bTBI can lead to traumatic vascular injury and vasospasm, and stimulation of vascular endothelial induces oxidative stress, which further aggravates vascular dysfunction^27–29^. Our results showed that some oxidative stress related genes- *sulfate-modifying factor 1* (*Sumf1*), *aldo-keto reductase family 1* (*Akr1e1*) and *tyrosine 3-monooxygenase activating protein theta* (*Ywhaq*), were down-regulated in endothelial-mural cells significantly. This is consistent with published studies on the relationship between oxidative stress and endothelial damage after TBI^30,31^. Further, we noted that a part of top DEGs from different cell types are involved in similar biological processes, including myelination, axon regeneration, and axon development (Fig. 3c,d). Consistently, these activities are known to be critical responses to brain injury^14,29,32^.

Furthermore, we built pseudo-time trajectories of all NSCs and identified 2 distinct branch points and 5 pseudo-time states using Monocle2((Fig. 3e). At branch point, cells must choose a certain gene expression program which determines their fate during development. These transcriptional programs are exclusive to each other. To reveal more detailed branch expression information, we showed the top six fate-specific genes that govern the cell fate decisions (Fig. 3f). At the same time, the top 50 genes that affect cell state and fate are presented in the heatmap (Fig. 3g).

Taken together, based on strict quality control and data filtering, we acquired a clean gene-cell expression matrix with clustering information. Gene expression and pseudo-time trajectories analysis further supported the quality of our sequencing data, and thus would be ideally suitable for downstream analysis and the future study on bTBI cell biology as well as the discovery of new diagnostic/ therapeutic targets.

## Usage Notes

This data-set can be used effectively (but not limited to) (1) to discover new cell type- specific markers of mouse SVZ, (2) to profile gene expression of each cell type in the mouse SVZ after bTBI, and to search for potential diagnostic/ therapeutic targets, (3) to predict the differentiation of NSCs in SVZ upon bTBI, and the genes governing this process (4) to study the cell- to- cell interactions in the SVZ after bTBI.

It should be noted that this sequencing data also has certain limitations. The background noise of snRNA-seq is relatively louder and more complicated than that of bulk RNA-seq. While many bio-informatics methods and tools have been developed to filter and analyze snRNA-seq data, new algorithms still need to be developed to ensure data repeatability.

Raw data is stored in the database in FASTQ format (uploaded in Gene Expression Omnibus).Subject accession number: GSE207078 for bTBI; GSE198074 for Sham. These data can be used as input for Cell Ranger pipeline or similar tools for analysis. The gene- barcode matrices file (Uploaded in Figshare^18,19^) can be processed using Seurat’s R package.

### Supplementary information

Dataset 1

## Data Availability

No special code was used for analysis of the current data-set. All of the analyses were done with the following open access programs: FastQC version 0.11.9. (https://github.com/s-andrews/FastQC). Cell Ranger version 3.1.0 (https://github.com/10XGenomics/cellranger). Seurat. Version 3.1.4 (https://satijalab.org/seurat).
