# Author Correction: Single-nucleus profiling of adult mice sub-ventricular zone after blast-related traumatic brain injury

**DOI:** 10.1038/s41597-023-02563-8

**Published:** 2023-10-02

**Authors:** Manrui Li, Xiameng Chen, Qiuyun Yang, Shuqiang Cao, Steven Wyler, Ruixuan Yuan, Lingxuan Zhang, Miao Liao, Meili Lv, Feng Wang, Yadong Guo, Jihong Zhou, Lin Zhang, Xiaoqi Xie, Weibo Liang

**Affiliations:** 1 Shanghai Key Lab of Forensic Medicine, Key Lab of Forensic Science, Ministry of Justice, Shanghai, 200000 China; 2 Department of Forensic Genetics, West China School of Basic Medical Sciences and Forensic Medicine, Sichuan University, Chengdu, 610041 China; 3 Department of Forensic Pathology and Forensic Clinical Medicine, West China School of Basic Medical Sciences and Forensic Medicine, Sichuan University, Chengdu, 610041 China; 4 Department of Internal Medicine, UT Southwestern Medical Center, Dallas, TX 75390 USA; 5 Sichuan University, Chengdu, 610041 China; 6 Department of Immunology, West China School of Basic Medical Sciences and Forensic Medicine, Sichuan University, Chengdu, 610041 China; 7 Department of Medical Oncology, Cancer Center, Sichuan University, Chengdu, 610041 China; 8 Department of Forensic Science, School of Basic Medical Sciences, Central South University, Changsha, Hunan 410013 China; 9 Army Medical University, Chongqing, 404000 China; 10 Department of Critical Care Medicine, Sichuan University, Chengdu, 610041 China

**Keywords:** White matter injury, Pathogenesis

Correction to: *Scientific Data* 10.1038/s41597-022-01925-y, published online 05 January 2023

In the Usage Notes section of the Data Descriptor, the last paragraph contained inaccurate information regarding the project accession numbers in the GEO database for the raw data, with the accession number “GSE198074” repeated twice. The correct details are as follows:

Raw data is stored in the database in FASTQ format (uploaded in Gene Expression Omnibus). Subject accession number: GSE207078 for bTBl; GSE198074 for Sham.

This has been updated in the pdf and HTML versions of the article.

