# Supplementary material for: Single-nucleus profiling of adult mice sub-ventricular zone after blast-related traumatic brain injury
Source: Sci Data. 2023 Jan 5;10:13. doi: 10.1038/s41597-022-01925-y (PMC9814753; doi:10.1038/s41597-022-01925-y)
Supplement: Supplementary file 1 — Dataset 1 [file 41597_2022_1925_MOESM1_ESM.pdf]

**Design Type(s)**	transcription profiling design sequence analysis objective • replicate design
**Measurement Type(s)**	transcription profiling assay
**Technology Type(s)**	single-nucleus RNA seqencing
**Factor Type(s)**	experimental condition:genotype
**Sample Chatacteristics(s)**	Mus musculus brain sub-ventricular zone cells
